# Neural correlates of hyperalgesia in the monosodium iodoacetate model of osteoarthritis pain

**DOI:** 10.1177/1744806916642445

**Published:** 2016-04-11

**Authors:** Maryam Abaei, Devi R Sagar, Elizabeth G Stockley, Clare H Spicer, Malcolm Prior, Victoria Chapman, Dorothee P Auer

**Affiliations:** 1 Radiological Sciences, Division of Clinical Neuroscience, University of Nottingham, Nottingham, UK; 2 Arthritis Research UK Pain Centre, University of Nottingham, Nottingham, UK; 3 School of Life Sciences, University of Nottingham, Nottingham, UK; 4 Medical Imaging Unit, School of Medicine, University of Nottingham, Nottingham, UK

**Keywords:** Hyperalgesia, pain fMRI, osteoarthritis model

## Abstract

**Background:**

The mechanisms driving osteoarthritic pain remain poorly understood, but there is increasing evidence for a role of the central nervous system in the chronification of pain. We used functional magnetic resonance imaging to investigate the influence of a model of unilateral knee osteoarthritis on nociceptive processing.

**Results:**

Four to five weeks post intra-articular injection of monosodium iodoacetate (MIA, 1 mg) into the left knee, Sprague Dawley rats were anesthetized for functional magnetic resonance imaging studies to characterize the neural response to a noxious stimulus (intra-articular capsaicin injection). In a two-arm cross-over design, 5 µM/50 µl capsaicin was injected into either the left knee (*n* = 8, CAPS-MIA) or right control knee (*n* = 8, CAPS-CON), preceded by contralateral vehicle (SAL) injection. To assess neural correlates of mechanical hyperalgesia, hindpaws were stimulated with von Frey hairs (8 g: MIA; 15 g: control knee, based on behavioral withdrawal responses). The CAPS-MIA group exhibited significant activation of the periaqueductal gray, unilateral thalamus and bilateral mensencephalon, superior-colliculus, and hippocampus, with no significant activation in the other groups/conditions. Capsaicin injection increased functional connectivity in the mid-brain network and mediodorsal thalamic nucleus, hippocampus, and globus pallidus, which was significantly stronger in CAPS-MIA compared to CAPS-CON groups. Mechanical stimulation of the hyperalgesic (ipsilateral to MIA knee) and normalgesic (contralateral) hindpaws evoked qualitatively different brain activation with more widespread brainstem and anterior cingulate (ACC) activation when stimulating the hyperalgesic paw, and clearer frontal sensory activation from the normalgesic paw.

**Conclusions:**

We provide evidence for modulation of nociceptive processing in a chronic knee osteoarthritis pain model with stronger brain activation and alteration of brain networks induced by the pro-nociceptive stimulus. We also report a shift to a medial pain activation pattern following stimulation of the hyperalgesic hindpaw. Taken together, our data support altered neural pain processing as a result of peripheral and central pain sensitization in this model.

## Background

The treatment of chronic pain, estimated to affect 19% of the adult European population,^1^ remains a clinical challenge. Osteoarthritis (OA) and arthritis combined are by far the most common cause of chronic pain in 42%;^1^ with a clear need for novel treatment strategies. In order to meet the therapeutic challenge of chronic arthritis/OA pain, a better understanding of the mechanisms of pain chronification is required. Animal models can provide vital insights into the neural mechanisms underpinning the experience of chronic pain, and in particular, the relative contribution of peripheral versus central sensitization. Indeed, the evaluation of pain behavior in rodent models of knee joint OA and their usefulness for the investigation of potential novel analgesic treatments for OA pain has been established in recent years.^2^ It is widely recognized that successful development of effective drug treatments for chronic pain depends on an interdisciplinary forward and backward translational approach based on advances in pain neurobiology and innovative pain models.^3,4^

Functional neuroimaging studies in OA patients with chronic pain provided important advances in characterizing the neural activation sites associated with spontaneous OA pain^5,6^ and those associated with evoked distal allodynia.^5,6^ Recent functional brain network analysis at rest, or during nociception, revealed that chronic OA pain is associated with functional reorganization,^7,8^ which is sensitive to treatment effects^9^ and has predictive power for placebo analgesia.^10^ The back-translation of these imaging findings to rodent models of OA pain has thus far only been explored in one model of OA pain, in which increased baseline functional connectivity in the nucleus accumbens and the ventroposterior lateral thalamus was shown in a surgical model of OA pain.^11^ Such studies provide the rationale for the further evaluation of the impact of altered sensory inputs from the knee joint on supraspinal networks in these rodent models of OA pain.

Assessing the functional brain connectome is a highly topical approach to gain an understanding of complex brain function and disorders in relation to underlying dynamic functional processes.^12^ Inference from functional connectivity studies, however, strongly depends on a well-controlled experimental design. This limitation is recognized in human studies but conceivably ever more pertinent in animal studies when investigating the poorly defined resting state or “baseline,” not least as the baseline is confounded by the need for anesthesia to ensure that subjects are still and welfare is maintained during imaging. To overcome this, and harness the advantages of functional connectome studies, this experimental approach needs to be combined with a well understood, and robust, pain stimulation during imaging. Evaluation of potential network changes in the supraspinal processing of sensory inputs from the knee joint which arise clinically can only be achieved by application of an additional stimulus during the imaging protocol in the established models of OA pain.

Recent work from our group has demonstrated increased activity in the sensory afferent fibers innervating the knee joint in the monosodium iodoacetate (MIA) model of OA pain, increased knee joint expression of transient receptor potential vanilloid 1 (TRPV1) in human OA knee samples, and that local knee joint blockade of TRPV1 with a selective antagonist attenuates OA pain behavior in the rodent model.^12^ These clinical and preclinical data support the notion that TRPV1 makes an important contribution to altered sensory afferent input from the OA knee joint and thus provide a rationale for the use of a TRPV1 ligand as an experimental tool to drive knee joint activation in this imaging study using a model of OA pain. Previously we have shown that intradermal injection of the TRPV1 ligand capsaicin (CAPS) increases blood oxygenation level dependent (BOLD) signal in brain regions associated with nociceptive processing, including thalamic, periaqueductal gray (PAG), mesencephalic nuclei, and the superior colliculus in anesthetized rats.^13^

The aim of the present study was to investigate the influence of established OA of the rat knee joint on the cerebral hemodynamic responses (using fMRI) to moderate noxious stimulation of the knee joint, and to mild noxious stimulation of the hindpaw. In the present study following characterization of pain behavior (weight bearing asymmetry and distal hindpaw mechanical withdrawal thresholds), rats were anesthetized and effects of intra-articular injection of the TRPV1 ligand CAPS, or saline, into the control or OA knee on regional brain activation were compared using the echoplanar BOLD-fMRI technique at 7 T, thus allowing the characterization of both brain activation and connectivity changes induced by TRVP1 activation. In addition, we used fMRI to investigate supraspinal central sensitization using mechanical stimulation of the ipsilateral and contralateral hindpaws, which, in analogy to human fMRI studies, was calibrated between OA and saline-injected knee to induce threshold withdrawal response.

## Methods

### Model of OA and pain behavior

Animal care was in accordance with the UK Animals (Scientific Procedures) Act of 1986. A total of 16 Sprague–Dawley rats (160–200 g at time of MIA injection; Charles River Laboratories, UK) were maintained on a 12:12 h light/dark schedule with food and water available ad libitum. An established model of OA pain was induced in 16 rats on Day 0 under isoflurane anesthesia (3%, 1 Lmin^−1^ O_2_). A single intra-articular injection of MIA (1 mg/50 μl; Sigma, UK) in saline was administered through the infra-patellar ligament of the left knee in all rats.^14^ The MIA model of OA pain has been widely used, and although it does not mimic the etiology of OA, it does mimic key clinical features of OA.^15^ Pain behavior was assessed for 22 days post injection after which rats were transferred to undergo imaging studies. Two separate measures of pain behavior were assessed: change in hindlimb weight distribution (weight bearing asymmetry), which is a behavioral correlate of hyperalgesia; and hindpaw mechanical withdrawal thresholds, which provides an index of central sensitization. Weight distribution was assessed using an incapacitance tester (Linton Instrumentation, UK) and was calculated as (weight on contralateral hind limb − weight on ipsilateral hind limb)/total weight on hind limbs × 100%. Paw withdrawal thresholds (PWTs) were assessed using a series of von Frey monofilaments (Semmes–Weinstein monofilaments of bending forces 1–15 g) applied in ascending order of bending force to the plantar surface as previously described.^2^ The PWT was the lowest weight of monofilament that elicited a withdrawal reflex. For analysis, a PWT of greater than 15 g was recorded as 26 g (the next consecutive von Frey weight in the series). Paw withdrawal data are non-parametric; therefore, non-parametric analysis was selected. Weight bearing data were analyzed using a D'Agostino and Pearson omnibus normality test (alpha = 0.05); data were not found to be normally distributed; therefore, non-parametric analysis were also selected. Data were compared to baseline values and analyzed using a Kruskall Wallis test with a Dunn’s multiple comparison post-hoc test.

### MRI protocol

fMRI acquisition was performed over four days, 28–32 days post MIA-injection, during which time pain behavior is stable.^2^ Rats were anesthetized with isoflurane (induction at ∼4%, maintenance at ∼2–2.5%), and temperature, breathing rate, and pulse oximetry was maintained in physiological range. An intra-articular cannula was inserted into each knee through the infrapatellar ligament using a 25G needle and secured in place.

Scanning was performed on a 7T Bruker MRI magnet using a volume transmit coil in conjunction with a receive-only head coil. Anatomical MRI data for the alignment of fMRI scanning were collected using a T2-weighted Rapid imaging with refocused echoes sequence in three orthogonal planes (30 slices, echo time (TE) = 23.6 ms, repetition time (TR) = 5000 ms, spatial resolution = 0.125 × 0.125 × 1.00 mm^3^). BOLD-fMRI data were acquired using a gradient-echo echo planar imaging sequence with TE = 23 ms, TR = 2000 ms, Matrix size = 64 × 64 × 17, spatial resolution = 0.46 × 0.46 × 1 mm^3^.

### Stimulation paradigm during fMRI acquisition

After anatomical images had been acquired and rats were physiologically stable, all rats underwent 10 min of mechanical stimulation in a 20 s on and 20 s off paradigm to either the ipsilateral or contralateral hindpaw (eight each, data from one control knee were discarded for technical reasons) using von Frey hairs selected on the basis of the behaviorally determined mechanical withdrawal threshold (average per group) prior to imaging. The group averaged mechanical withdrawal threshold of the MIA (left) hind paw was 8 g von Frey hair vs. 15 g von Frey hair for the control (right) hind paw.

**Figure 1.**
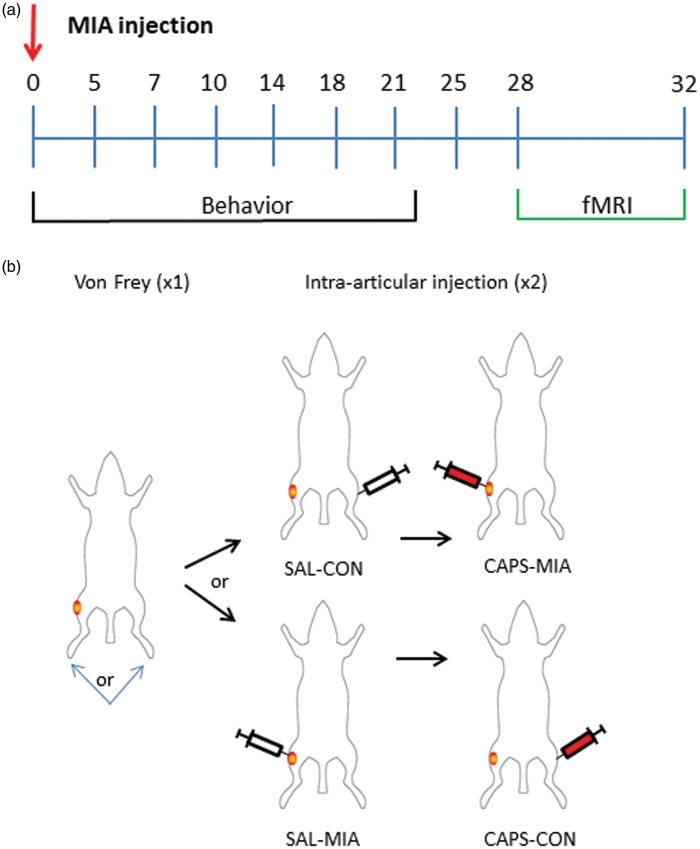
Study design: (a) across 32 day time period; (b) stimulation protocol during fMRI: 10 min block design (20 s on/20 s off) mechanical pain stimulation using 8 (or 15) g von Frey to the left (or right [L/R]) hindpaw and then 60 min single cross-over fMRI consisting of baseline, at 10 min, intra-articular (i.a.) injection of 50 µl saline (SAL) to MIA or control knee (CON), and at 25 min contralateral intra-articular injection of CAPS (50 µl CAPS).

For the noxious stimulus (CAPS) fMRI scan, a total of 60 min scanning was performed (Figure 1): 10 min baseline (baseline resting state); 15 min scan following intra-articular injection of 50 μl of vehicle into either left or right knee (vehicle control state); 35 min scan following intra-articular injection of 50 μl of 5 μM CAPS into the opposite knee (CAPS-induced pain state). Half of the OA rats received the injection of vehicle to the control (right) knee and CAPS to the MIA (left) knee (MIA-CAPS), the other half received vehicle to the MIA knee and CAPS to the control knee (“CON-CAPS”). This design was selected to minimize possible carry over effects between the von Frey stimulus and the CAPS condition. In addition, we balanced the side of von Frey hind paw stimulation between the groups, half of MIA-CAPS group had previously undergone ipsilateral or contralateral von Frey stimulation, to further mitigate against an order effect.

### fMRI analysis

For easier interpretation of results, activation maps were generated from flipped data. Before any pre-processing, data from rats receiving CAPS in their right knee (CON-CAPS) were rotated around the y axis to swap the left and right hemispheres; hence, figures can be interpreted as though all rats received CAPS into the left knee. In addition, to study possible laterality effects of pain processing as suggested in a recent human pain fMRI meta-analysis,^16^ we also analyzed un-flipped data.

For pain threshold (von Frey) activation maps, model-based fMRI data processing was carried out using FEAT (fMRI Expert Analysis Tool, Version 5.98; University of Oxford, Oxford, UK), which is part of FSL (FMRIB Software Library; www.fmrib.ox.ac.uk/fsl). Specifically, data were corrected for head motion using motion correction FMRIB’s linear image registration tool, removal of the skull and brain extraction, spatial smoothing at standard 1 mm and 3 mm to account for expected low activation height in pain threshold studies of anesthetized rodents. There were no differences in head motion parameters in absolute (relative to the reference volume) (MIA-CAPS [0.24 ± 0.01 mm] vs. CON-CAPS [0.20 ± 0.08 mm, t-test *p* = 0.42]) or relative displacement (relative to next volume) (MIA-CAPS [0.07 ± 0.01 mm] vs. CON-CAPS [0.07 ± 0.01 mm, t-test *p* = 0.7]) between the study groups. Maximum absolute (0.37 mm) and relative (0.11 mm) head motion were below the voxel size in all subjects.

Following pre-processing, time-series statistical analyses using FMRIB’s improved linear model with local autocorrelation corrections and block design were used for first-level analyses; to create statistical parametric maps of the functional data, six motion parameters were used as variables of no interest. Following the first-level analysis, group analysis was performed using FMRIB’s Local Analysis of Mixed Effects (statistical model of mixed-effects variance) to estimate random effects and variances between subjects. t-tests were performed to determine the differences in activation within and between groups (*Z* > 2.3 and a corrected cluster significance threshold of *p* ≤ 0.05).

### Model-free analysis

To assess the effects of intra-articular injection of CAPS on functional brain connectivity, and how this may be modulated by MIA-induced TRPV1 sensitization, we compared functional brain networks (so-called resting state networks [RSN]) before and after intra-articular injection of CAPS and between groups (MIA vs. control knee injection).

Data were pre-processed and analyzed using FSL, including motion correction using motion correction FMRIB’s linear image registration tool and removal of non-brain structures with Brain Extraction Tool. Gaussian spatial smoothing was applied with a full width half maximum of 1 mm. ICA was carried out using FSL4.1.4 MELODIC software implementing probabilistic independent component analysis (PICA).^17^ A multisession temporal concatenation tool in MELODIC was used to perform PICA related pre-processing and data conditioning in the group analysis setting.

Spatial ICA using 35 IC maps was applied to detect RSNs. A rat atlas template^18^ was used to identify the anatomical characteristics of the resulting PICA maps. RSNs included were thalamus, motor cortex, sensory cortex, and PAG. ICs were subjected to dual-regression analysis to determine any spatial differences for individual networks between the three conditions (post CAPS vs. vehicle, post CAPS vs. baseline, and vehicle vs. baseline) and the higher level between group (CAPS injection sites) difference of the contrast for baseline vs. post-CAPS. Comparison between the two CAPS injection site groups before any injection served as a further validation contrast as the groups and conditions should have been identical. A statistical analysis was performed using FSL randomize nonparametric permutation testing, with 5000 permutations and t-test for between group differences and paired t-test for within group differences using the threshold free cluster enhancement to determine significance at corrected *p* ≤ 0.05 level.

## Results and discussion

### Pain behavior

**Figure 2.**
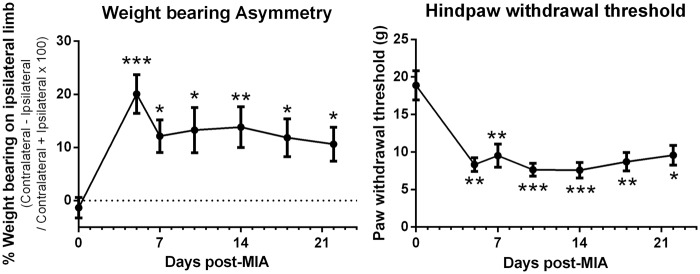
Intra-articular injection of MIA significantly increased weight bearing asymmetry and decreased hind paw withdrawal thresholds on the ipsilateral side compared to baseline. Data are expressed as mean ± SEM (*n* = 16 rats). Statistical analysis was performed using a Kruskall Wallis test with a Dunn’s multiple comparison test. *p < 0.05, **p < 0.01, *** *p* < 0.001 compared to baseline.

Intra-articular injection of MIA was associated with significant decreases in hindlimb weight bearing and mechanical hindpaw withdrawal thresholds on the ipsilateral side compared to baseline values (Figure 2). These changes were present from Day 5 post-MIA injection and were maintained throughout the duration of the study.

### CAPS-induced brain activation in the MIA model

**Figure 3.**
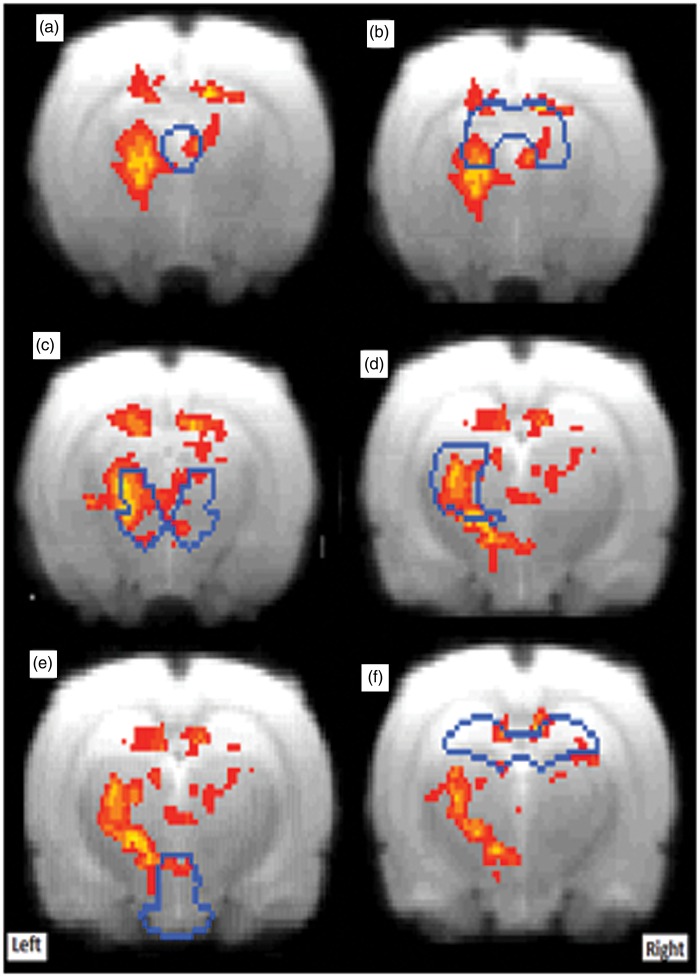
Group mean fMRI activation (red–yellow, *p* < 0.05 corrected) for MIA-CAPS (CAPS injection into MIA knee, *n* = 8), overlaid on average T2-structural image. Blue lines indicate the borders for: (a) PAG, (b) Superior-Colliculus, (c) Mesencephalic region, (d) Thalamus, (e) Hypothalamus, and (f) Hippocampus.

Using the stimulation paradigm outlined in Figure 1, intra-articular injection of 50 μl of CAPS into the MIA-treated knee induced significant activation in the following brain areas: PAG, unilateral thalamus and bilateral mesencephalon, superior colliculus, and hippocampus (Figure 3), but injection of 50 μl of CAPS into the control knee did not induce significant brain activation. However, despite the selective CAPS effect when injected into the MIA pre-treated knee, with higher level contrast analysis, there were no significant differences between the two groups at the corrected level likely reflecting a type II error. Injection of saline into control or MIA knee had no effect on brain activity (no significant condition or group effect).

### CAPS and MIA effects on functional brain networks

Model free independent component analysis of the concatenated fMRI data across both baseline and post CAPS conditions for all 16 rats detected 35 independent components (IC). As expected for the level of anesthesia, IC maps identified mainly short range RSN corresponding to morphological structures such as the thalamus, primary motor cortex, somatosensory cortex, hippocampus, and internal capsule/sensory cortex.

**Figure 4.**
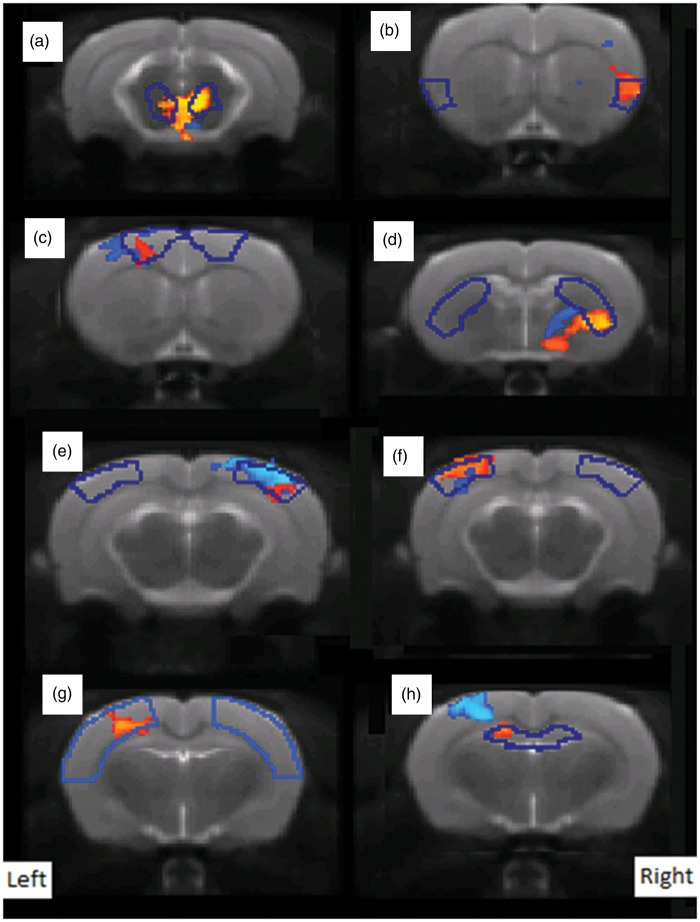
CAPS-induced increased functional connectivity map in MIA-CAPS group (red–yellow) and in CON-CAPS group (blue-light) in axial view, overlaid on T2-weighted template (*p* < 0.05 corrected). The blue line indicates the structural borders of (a) Mesencephalic Region, (b) Cortex Insular, (c) Primary Motor Cortex, (d) Caudate-Putamen, (e, f) Cortex Parietal Association, (g) Cortex Somatosensory, and (h) Hippocampus Anterior Dorsal.

We then compared baseline versus CAPS for each group showing that injection of CAPS into the MIA knee induced significantly increased functional connectivity of several RSNs, including the thalamus, primary motor cortex, somatosensory and frontal sensory cortices, and hippocampus (red–yellow, Figure 4); no functional connectivity decreases were noted. CAPS also induced significant functional connectivity increases in the sensory motor cortex, parietal association cortex, and hippocampus in the CON-CAPS group (blue, Figure 4), again no significant decreases in functional connectivity were noted. The pattern of functional connectivity increased in MIA-CAPS versus CON-CAPS groups was only partially overlapping.

**Figure 5.**
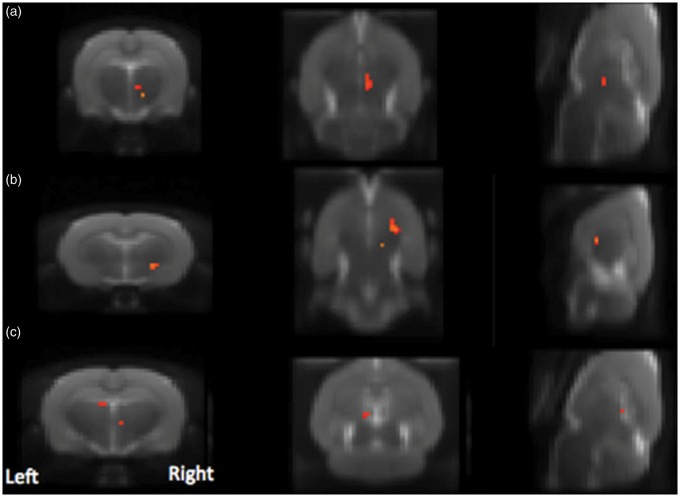
Intra-articular injection of CAPS induced an increase of functional connectivity in the mediodorsal thalamic nucleus (a), Hippocampus (b), and Globus Pallidus (c) in MIA-CAPS group compared to CON-CAPS group; corrected *P* < 0.05.

Between groups analysis using dual regression identified an exclusive differential effect of intra-articular injection of CAPS into the MIA knee versus control knee on the mid-brain network. Specifically, we report an increased functional connectivity between mensencephalon-mediodorsal thalamic nucleus and hippocampus and globus pallidus in the MIA-CAPS group, compared to the MIA-CON group (Figure 5(a) and (c)).

As expected, we found no difference in RSN functional connectivity between the two MIA injection groups at baseline. Also, the RSN functional connectivity after vehicle injection in MIA or control knee did not significantly differ from the baseline condition.

### Functional MRI of remote hyperalgesia of the hindpaw

**Figure 6.**
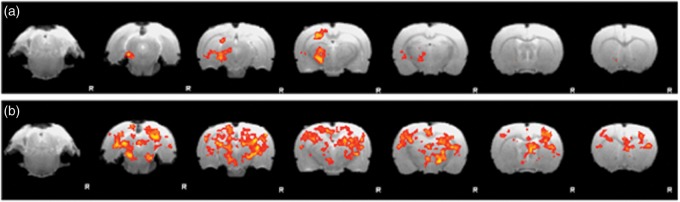
Activation maps (False discovery rate corrected, *p* < 0.05) evoked by von Frey pain stimulation of the hindpaw with a threshold hair: (a) ipsilateral to the control (left) knee (15 g von Frey hair) and (b) ipsilateral to the MIA (right) knee (8 g von Frey hair). Von Frey weights were chosen as the respective minimum force needed to elicit withdrawal in awake rats.

Mechanical stimulation of the hindpaw did not evoke any detectable brain activation between and within the groups when a 1 mm spatial smoothing was employed. However, using a 3-mm spatial smoothing revealed a unilateral activation of the contralateral thalamus, brainstem, and hippocampus following stimulation of the control (left) hindpaw with a 15-g von Frey hair. This stimulus was shown to be the threshold hair to elicit a withdrawal response in awake rats (Table 1, Figure 6(a)). In contrast, stimulation of the MIA (right) hindpaw with a 8-g von Frey hair (withdrawal threshold in the awake rats) was associated with a widespread and bilateral activation pattern, including additional regions of activation in the brainstem, bilateral thalami, hippocampi and parietal association cortices, and medial prefrontal cortex/anterior cingulate (Table 1, Figure 6(b)). However, these apparent differences in activation extent between the groups were not confirmed by higher level between group contrast analysis, when corrected for multiple tests. Voxel-based higher level contrasts are known to be prone to type II error.

**Table 1.** Localization of activation in control knee von Frey hindpaw stimulation according to Paxinos Atlas.^18^

		*Coordinate*		
*Location*	*Zscore*	*X*	*Y*	*Z*
Control knee von Frey hindpaw stimulation				
Thalamus, dorsolateral	2.94	−26.7	−40	−53.4
Hippocampus, posterior, ventral part	2.89	−45.2	−56	−57.5
Mesencephalic region	2.81	20.5	−56	−57.5
MIA knee von Frey hindpaw stimulation				
Hypothalamus, lateral	3.37	18.5	−24	781
Thalamus, dorsolateral	3.32	22.6	32	37
Hippocampus, subiculum	3.29	37	−64	−34.9
Pedunculopontine tegmental nucleus	3.28	−18.5	−80	−74
Cortex, somatosensory	3.26	57.5	48	−34.9
Mesencephalic region	3.46	−12.3	−56	−76
Cortex, cingulated	2.91	−2.05	16	−36.9

### Neural correlates of pain sensitization in the MIA-OA model

Our study demonstrates neural correlates of peripheral and central sensitization in the MIA-induced knee OA pain model in rats. Intra-articular injection of CAPS into the MIA-treated knee induced bilateral subcortical and hippocampal activation, but no effect was seen using the same injection (stimulus locked) in the control knee (Table 2). Intra-articular injection of CAPS into both MIA and control knee increased functional connectivity in several networks, but we found a significantly greater increase in mesencephalic functional connectivity with injection of CAPS into the MIA knee. Remote hyperalgesia was tested using behaviorally locked mechanical von Frey stimulation of the hindpaw at the respective withdrawal threshold. Despite the weight of von Frey stimulation in the MIA group being half of that required for the control group, we showed widespread bilateral subcortical and cortical brain activation following von Frey stimulation in the MIA group compared to unilateral activation of the sensorimotor network during von Frey stimulation of the control hindpaw.

**Table 2.** Summary of nociceptive brain* activation foci and changes in functional connectivity within and between experimental groups and conditions. NA: Not Applicable*.

Contrasts	Anatomical Structure	
	Activation (corr. P < 0.05)	FC (corr. p < 0.05)
MIA-CAPS > CON-CAPS	Not significant	Mediodorsal thalamic nucleus, Hippocampus, and Globus Pallidus
MIA-CAPS > Baseline	PAG, Superior-Colliculus, Mesencephalic region, Thalamus, Hypothalamus Medial, Hippocampus.	Mesencephalic Region, Cortex Insular, Primary Motor Cortex, Caudate-Putamen, Thalamus, Cortex Parietal Association, Cortex Somatosensory, Hippocampus
CON-CAPS > Baseline	Not Significant	Primary Motor Cortex, Caudate-Putamen, Cortex Parietal Association, Cortex Somatosensory
MIA > CON Von-Frey	Not Significant	NA
MIA hindpaw: 8 gr Von-Frey	PAG, Mesencephalic region, Hippocampus, thalamus, and cortex parietal association	NA
CON hinpaw: 15 g Von-Frey	Hippocampus posterodorsal/subiculum and cortex parietal association	NA

CAPS: capsaicin; PAG: periaqueductal gray; CON: control; MIA: monosodium iodoacetate; FC: functional connectivity.

One of the aims of our study was to investigate whether MIA-induced sensitization in the chronic knee OA pain model was characterized by differential brain activation, which may reflect the behavioral hyperalgesia demonstrated herein and previously. We report brain activation in subcortical and hippocampal brain areas specific to injection of CAPS into the MIA joint, predominantly in the brainstem and contralateral thalamus. Despite different models and imaging methods used, our data are largely consistent with previous preclinical imaging studies reporting subcortical hyperactivation in hyperalgesic states: increased thalamic and PAG activation was detected in a pancreatic inflammation model^19^; increased hypothalamus and brainstem activation in post-surgical mechanical hyperalgesia^20^; increased thalamic activation in colonic hypersensitivity^21^; and increased PAG activation following hindpaw injection of CAPS.^13^ We did not observe significant primary somatosensory cortex activation, which was reported in earlier fMRI studies following 25 μl and 75 μl injection of CAPS into the dosal forepaw^22^ and ankle, respectively.^23^ Sensory cortical hyperactivation may be a hallmark of mechanical allodynia as shown in a spinal cord injury neuropathic pain model.^24^ The lack of observation of CAPS-induced activation of the somatosensory and anterior cingulate cortices is noteworthy given their common co-activation in human pain studies, and previous preclinical CAPS injection studies.^22,23^ This may be due to protocol differences related to the scan acquisition, dose and site of injection, and importantly anesthesia protocol. The cortical somatosensory representation of both dorsal forepaw and ankle^22,23^ can be expected to be larger than that of the knee joint. There are also differences in field strength (9.4 vs. 7T) and imaging protocol (standard vs. higher temporally resolved echoplanar scans), but the likely main difference is the use of alpha-chloralose anesthesia in the prervious studies vs. isoflurane in the present study.

Since our data were collected under anesthesia, brain activation induced by our noxious stimulus can only be ascribed to enhanced nociception rather than pain perception, which is precluded by the experimental conditions. All experiments were undertaken under spontaneous isoflurane inhalation anesthesia at a maintenance dose of 2%–2.5% to minimize arousal. Under this anesthetic regimen, we observed no BOLD activation upon intra-articular injection of CAPS into the control knee. This finding is likely related to the depth of anesthesia as we cannot exclude that this dose of isoflurane had some analgesic effect despite the lack of analgesia induced by subanesthetic doses of isoflurane.^25^ Nevertheless, limbic activation and the lack of detectable cortical activation following intra-articular injection of CAPS into the MIA knee is broadly consistent with multivariate pattern analysis of brain activity at rest in a neuropathic pain model as studied in awake rats undergoing micro-positron emission tomography with [18F]fluorodeoxyglucose.^26^ In this previous study, several brain regions contributed to an accurate distinction of the spinal nerve ligation model with mechanical hypersensitivity from sham rats; specifically, secondary somatosensory cortex and other cortical regions showed decreased brain activity while mainly subcortical and (para-)limbic regions displayed increased activity. Several of the brain regions with positive loading for the discrimination model overlapped with the evoked brain activity we identified following von Frey stimulation of the hyperalgesic hindpaw. Taken together, our experimental protocol had limited sensitivity to normalgesia but adequate sensitivity to the hyperalgesic CAPS and von Frey condition.

Interestingly, we identified more pronounced alterations in the functional connectivity than amplitude changes during the pronociceptive state. RSN changes were noted following intra-articular injection of CAPS into both the MIA and control normalgesic knee. Despite the presence of anesthesia, group IC analysis (ICA) identified most known RSNs including the thalamus, hippocampus, parietal cortex, motor somatosensory cortices in line with previous studies in anesthetized rats.^27,28^ We also showed that hyperalgesia modulated the functional connectivity changes following intra-articular injection of CAPS. CAPS injection into the MIA knee induced a significantly greater functional connectivity increase in the mediodorsal thalamic nucleus, hippocampus, and globus pallidus, compared to injection of CAPS into the control knee. These data provide evidence that peripheral and spinal pain sensitization not only augment nociceptive drive and increase subcortical brain activation but also increase the functional brain connectivity response within subcortical and limbic networks. This is generally in line with the increased recognition of the power of RSN analysis to understand altered brain function.^12^ In a surgical model of OA (medial meniscal tear), increased functional connectivity compared to sham operated rats was reported at baseline, which was modulated by cyclooxygenase-2 inhibition.^11^ Similar to our study, functional connectivity changes were reported in the presence of 2% isoflurane. In contrast to the stimulation-free study design in Upadhyay,^11^ we observed differences in BOLD activation and functional connectivity following intra-articular injection of CAPS, which we interpret to represent the up-regulation of the nociceptive drive and hence more directly reflecting central sensitization. Although we cannot rule out the occurrence of cortical descending pain augmentation in this model, these effects would be expected to be symmetric and hence not detectable in our within group study design. Thus, the observed within subject differences in brain activation patterns likely reflect up-regulation of the stimulus input due to either peripheral hyperalgesia and/or central sensitization.

Strengths of our experimental design include the well-controlled within subject design using a robust intra-articular noxious stimulation in a well-characterized rodent model of chronic OA pain. This allowed us to directly compare combined BOLD amplitude and functional connectivity changes during an evoked pronociceptive state in the normalgesic and hyperalgesic knee. To address the limitation of stimulus-locked injection of CAPS, we also deployed a behavioral locked nociceptive design to characterize brain activity changes reflecting brain plasticity associated with central sensitization. Using a mechanical stimulus, based on that required to evoke a withdrawal reflex response of the hyperalgesic versus non-hyperalgesic remote hindpaw in the awake rat, we provide evidence for the central activation patterns associated with central sensitization alone without the confounds of using differing levels of nociceptive peripheral input. We report unilateral predominantly sensory network activation following stimulation of the non-hyperalgesic hindpaw, and widespread bilateral activation of the sensory and midline emotional networks following stimulation of the hyperalgesic hindpaw. To the best of our knowledge, behavioral locked nociception has not previously been reported in imaging studies using rodent models of chronic pain. Human fMRI studies of chronic OA pain also typically compare stimuli or tasks that induce differential pain in patients and controls.^29^ The common inference that differential brain activation patterns in such experimental designs represent group differences in central sensitization, rather than processing of different levels of pain intensity, remains speculative. The main limitation of our study is our limited sensitivity to detect brain activation during normalgesic conditions, and therefore lack of ability to identify significance between condition BOLD amplitude changes. Despite distinctly different activation BOLD amplitude patterns between experimental groups, only the functional connectivity changes showed significant between condition differences.

## Conclusion

We report that enhanced TRPV1-mediated nociceptive signaling in a chronic MIA model of OA pain is associated with enhanced mesencephalic functional connectivity and subcortical BOLD activation. Using a behavioral locked nociceptive stimulus, we provide evidence for a change from unilateral sensory nociception to widespread bilateral brain activation under conditions of central sensitization in this model of OA pain.
